# Supplementary material for: Open-source personal pipetting robots with live-cell incubation and microscopy compatibility
Source: Nat Commun. 2022 May 30;13:2999. doi: 10.1038/s41467-022-30643-7 (PMC9151679; doi:10.1038/s41467-022-30643-7)
Supplement: Supplementary file 2 — Reporting Summary [file 41467_2022_30643_MOESM2_ESM.pdf]

nature portfolio

Corresponding author(s): Timm Schroeder
Philip Dettinger

Last updated by author(s): 12.04.2022

Reporting Summary

Nature Portfolio wishes to improve the reproducibility of the work that we publish. This form provides structure for consistency and transparency in reporting. For further information on Nature Portfolio policies, see our Editorial Policies and the Editorial Policy Checklist.

Statistics

For all statistical analyses, confirm that the following items are present in the figure legend, table legend, main text, or Methods section.

n/a	Confirmed	
☐	☒	The exact sample size (n) for each experimental group/condition, given as a discrete number and unit of measurement
☐	☒	A statement on whether measurements were taken from distinct samples or whether the same sample was measured repeatedly
☐	☒	The statistical test(s) used AND whether they are one- or two-sided<br>*Only common tests should be described solely by name; describe more complex techniques in the Methods section.*
☒	☐	A description of all covariates tested
☐	☒	A description of any assumptions or corrections, such as tests of normality and adjustment for multiple comparisons
☐	☒	A full description of the statistical parameters including central tendency (e.g. means) or other basic estimates (e.g. regression coefficient) AND variation (e.g. standard deviation) or associated estimates of uncertainty (e.g. confidence intervals)
☐	☒	For null hypothesis testing, the test statistic (e.g. F, t, r) with confidence intervals, effect sizes, degrees of freedom and P value noted<br>*Give P values as exact values whenever suitable.*
☒	☐	For Bayesian analysis, information on the choice of priors and Markov chain Monte Carlo settings
☒	☐	For hierarchical and complex designs, identification of the appropriate level for tests and full reporting of outcomes
☒	☐	Estimates of effect sizes (e.g. Cohen's d, Pearson's r), indicating how they were calculated

Our web collection on statistics for biologists contains articles on many of the points above.

Software and code

Policy information about availability of computer code

Data collection	MATLAB 2020, BD FACS Diva<br>https://github.com/CSDGroup/PHIL
Data analysis	Flowjo 10, R 4.1.2 (R-Project), MATLAB 2020

For manuscripts utilizing custom algorithms or software that are central to the research but not yet described in published literature, software must be made available to editors and reviewers. We strongly encourage code deposition in a community repository (e.g. GitHub). See the Nature Portfolio guidelines for submitting code & software for further information.

Data

Policy information about availability of data

All manuscripts must include a data availability statement. This statement should provide the following information, where applicable:
- Accession codes, unique identifiers, or web links for publicly available datasets
- A description of any restrictions on data availability
- For clinical datasets or third party data, please ensure that the statement adheres to our policy

Source data are provided with this paper.

Field-specific reporting

Please select the one below that is the best fit for your research. If you are not sure, read the appropriate sections before making your selection.

- [x] Life sciences
- [ ] Behavioural & social sciences
- [ ] Ecological, evolutionary & environmental sciences

For a reference copy of the document with all sections, see nature.com/documents/nr-reporting-summary-flat.pdf

Life sciences study design

All studies must disclose on these points even when the disclosure is negative.

Sample size	Sample sizes were selected in order to ensure reproducibility.
Data exclusions	Data was only excluded in the event of obvious mechanical failures (e.g. stalled pumps, loose screws, etc.).
Replication	All findings contained in this manuscript were successful replicated.
Randomization	For "manual" classification, time series were assigned to the predefined categories non-responsive, sustained, transient, oscillatory and unclear/outlier by the experimenter. Time series were chosen randomly and displayed without any meta data (e.g. stimulation information) to the experimenter by an algorithm implemented in R to assure that there was no classification bias. All series of all experiments of the GMP data set were classified in a single session.
Blinding	For "manual" classification, time series were assigned to the predefined categories non-responsive, sustained, transient, oscillatory and unclear/outlier by the experimenter. Time series were chosen randomly and displayed without any meta data (e.g. stimulation information) to the experimenter by an algorithm implemented in R to assure that there was no classification bias. All series of all experiments of the GMP data set were classified in a single session.

Reporting for specific materials, systems and methods

We require information from authors about some types of materials, experimental systems and methods used in many studies. Here, indicate whether each material, system or method listed is relevant to your study. If you are not sure if a list item applies to your research, read the appropriate section before selecting a response.

Materials & experimental systems

n/a	Involved in the study	
[ ]	[x]	Antibodies
[ ]	[x]	Eukaryotic cell lines
[x]	[ ]	Palaeontology and archaeology
[ ]	[x]	Animals and other organisms
[x]	[ ]	Human research participants
[x]	[ ]	Clinical data
[x]	[ ]	Dual use research of concern

Methods

n/a	Involved in the study	
[x]	[ ]	ChIP-seq
[ ]	[x]	Flow cytometry
[x]	[ ]	MRI-based neuroimaging

Antibodies

Antibodies used

Immunostaining Antibodies:
Chicken-anti GFP (Aves, AB_2307313)
Goat anti-Collagen 1 IgG (Southern Biotech, 1310-01)
Rat anti-CD41 IgG (eBioscience, 16-0411-85)
Mouse anti-GFAP IgG (R&D Systems, MAB2594)
Alexa Fluor 647 donkey anti-goat IgG (Invitrogen, A-21447)
Alexa Fluor 555 donkey anti-rat IgG (Invitrogen, A-48270)
Alexa Fluor 546 donkey anti-mouse IgG (Invitrogen, A-10036)
Alexa Fluor 488 donkey anti-chicken IgG (Invitrogen, AB_2340375)

Biotinylated lineage antibodies:
CD3ε (Invitrogen, 145-2C11)
CD19 (Invitrogen, eBio1D3)
TER-119 (Invitrogen, TER-119)
B220 (Invitrogen, RA3-6B2)
Ly-6G (Invitrogen, RB6-8C5)
CD11b (Invitrogen, M1/70)

FACS antibodies:
CD16/32-PerCp-Cy5.5 (Biolegend, Clone 93)
Sca1-Pacific Blue (Biolegend, Clone D7)
cKit-BV510 (Biolegend, Clone 2B8)
CD150-BV650 (Biolegend, Clone TC15-12F12.2)
streptavidin-BV711 (BD Biosciences)
CD34-eFluor660 (Invitrogen, Clone RAM34)
CD48-APCeFl780 (Invitrogen, Clone HM48-1)
CD16/32-APCCY7 (Biolegend, Clone 93)
Sca1-PacBlue (Biolegend, Clone D7)
cKit-BV711 (BD Biosciences, Clone 2B8)
CD150-BV650 (Biolegend, Clone TC15-12F12.2)
streptavidin-BV570 (Biolegend)
CD41-PerCPeFL710 (eBioscience, Clone MWReg30)
CD105-APC (Biolegend, Clone MJ7/18)

Validation

All antibodies used were previously optimized and validated and compared to IgG controls or secondary-only controls (1). The source of antibodies used is described in the materials and methods.

1. Coutu, D., Kokkaliaris, K., Kunz, L. et al. Three-dimensional map of nonhematopoietic bone and bone-marrow cells and molecules. Nat Biotechnol 35, 1202–1210 (2017). https://doi.org/10.1038/nbt.4006

Eukaryotic cell lines

Policy information about cell lines

Cell line source(s)

R1 WT cells were obtained from Konstantinos Anastassiadis at the Transgenic Core Facility of the Technical University of Dresden.

Authentication

None of the cell lines used were authenticated.

Mycoplasma contamination

All cell lines tested negative for mycoplasma contamination.

Commonly misidentified lines
(See ICLAC register)

Our R1 cell line have not been listed as misidentified.

Animals and other organisms

Policy information about studies involving animals; ARRIVE guidelines recommended for reporting animal research

Laboratory animals

Experiments were conducted with 12-16-weeks old male GATA2VENUS and GFP-p65/H2B-mCHERRY mice. Transgenic mice were previously published. Ahmed, N. et al. A Novel GATA2 Protein Reporter Mouse Reveals Hematopoietic Progenitor Cell Types. Stem Cell Reports 15, 326–339 (2020).

Wild animals

This study did not involve wild animals.

Field-collected samples

This study did not involve field collected samples.

Ethics oversight

Animal experiments were approved according to Institutional guidelines of ETH Zurich and Swiss Federal Law by the veterinary office of Canton Basel-Stadt, Switzerland (approval no. 2655).

Note that full information on the approval of the study protocol must also be provided in the manuscript.

Flow Cytometry

Plots

Confirm that:

- [x] The axis labels state the marker and fluorochrome used (e.g. CD4-FITC).
- [x] The axis scales are clearly visible. Include numbers along axes only for bottom left plot of group (a 'group' is an analysis of identical markers).
- [x] All plots are contour plots with outliers or pseudocolor plots.
- [x] A numerical value for number of cells or percentage (with statistics) is provided.

Methodology

Sample preparation

Primary cells were isolated and sorted as previously described. In brief, femurs, tibiae, coxae, and vertebrae were isolated and crushed in PBS (2% FCS, 2 mM EDTA) and filtered through a 40-µm nylon mesh. Erythrocytes were lysed for 3 min on ice in ACK lysing buffer (Lonza), stained with biotinylated lineage antibodies against CD3ε (145-2C11), CD19 (eBio1D3), TER-119 (TER-119), B220 (RA3-6B2), Ly-6G (RB6-8C5) and CD11b (M1/70), and labelled with streptavidin-conjugated magnetic beads

(Roti-MagBeads, Roche) before immuno-magnetic depletion. For isolation of GFP-p65 GMPs, cells were stained with CD16/32-PerCp-Cy5.5 (Biolegend, Clone 93), Sca1-Pacific Blue (Biolegend, Clone D7), cKit-BV510 (Biolegend, Clone 2B8), CD150-BV650 (Biolegend, Clone TC15-12F12.2), streptavidin-BV711 (BD Biosciences), CD34-eFluor660 (Invitrogen, Clone RAM34) and CD48-APCeFl780 (Invitrogen, Clone HM48-1). For isolation of GATA2VENUS myeloid progenitors, cells were stained with CD16/32-APCCY7 (Biolegend, Clone 93), Sca1-PacBlue (Biolegend, Clone D7), cKit-BV711 (BD Biosciences, Clone 2B8), CD150-BV650 (Biolegend, Clone TC15-12F12.2), streptavidin-BV570 (Biolegend), CD41-PerCPeFL710 (eBioscience, Clone MWReg30) and CD105-APC (Biolegend, Clone MJ7/18). Cells were stained for 90 min on ice and sorted using a BD FACS Aria I or III with 70-µm nozzle, single-cell purity mode and sorting purities ≥ 98%.

Instrument	FACS ARIA III (BD Biosciences)
Software	BD FACS Diva and Flowjo 10
Cell population abundance	Optimal sorting settings and purity were determined first by sorting and reanalysis of Lineage negative cKit high cells. Sorting purity was ≥ 98%.
Gating strategy	The FACS gating scheme was used according to previously published protocols and included. FSC/SSC gates were used prior to analysis to exclude dead cells, doublets and leukocytes and to annotate hematopoietic stem and progenitor cells. The positive and negative gates were set based on relative marker expression between different populations. FACS gating scheme used for isolation of myeloid progenitors from GATA2VENUS mice in Figure 3B.<br>Sorting strategy: Lineage negative SCA1 negative cKIT high CD41 negative CD105 negative CD150 negative

☒ Tick this box to confirm that a figure exemplifying the gating strategy is provided in the Supplementary Information.
