# Supplementary material for: Open-source personal pipetting robots with live-cell incubation and microscopy compatibility
Source: Nat Commun. 2022 May 30;13:2999. doi: 10.1038/s41467-022-30643-7 (PMC9151679; doi:10.1038/s41467-022-30643-7)
Supplement: Supplementary file 3 — Description of Additional Supplementary Files [file 41467_2022_30643_MOESM3_ESM.pdf]

Title: Supplementary Movie 1:
Description: PHIL pipetting in a 96 well plate.
PHIL utilizes 5-bar planar geometry to move pipet tips from well to well in a 96 well plate and dispenses food dye into targeted wells.

Title: Supplementary Movie 2:
Description: PHIL assembly in 2.5 h.

Title: Supplementary Movie 3:
Description: PHIL pipetting of a 20 s pulse.
100 µL PBS is replaced with 100 µL FITC before being replaced with 100 µL PBS. Imaged every 0.5 s (mm:ss timestamp).

Title: Supplementary Movie 4:
Description: PHIL pipetting of sequential 15 s pulses to generate oscillations.
To generate oscillating FITC stimulation patterns, the all 100 µL of a 96 well plate well are sequentially changed from PBS to FITC to PBS every 15 s. Imaged every 0.5 s (mm:ss timestamp).

Title: Supplementary Movie 5:
Description: PHIL pipetting of 2 h oscillations.
100 µL PBS and FITC oscillations in a 96 well plate are maintained over 48 h. Imaged every 10 min (hh:mm timestamp).

Title: Supplementary Movie 6:
Description: GMPs are unaffected by fluid flow.
GMPs were automatically washed with 50 µL/s flow. No cell displacement was observed. Imaged every 9 min (hh:mm timestamp).

Title: Supplementary Software
Description: This compressed file contains all assembly and operation instructions as well as 3D printable models and robot control software.
