# Supplementary material for: Open-source personal pipetting robots with live-cell incubation and microscopy compatibility
Source: Nat Commun. 2022 May 30;13:2999. doi: 10.1038/s41467-022-30643-7 (PMC9151679; doi:10.1038/s41467-022-30643-7)

PHIL Operation

1. **Operate PHIL**
   a. **Start GUI**
      i. Open latest GUI version.
      ii. Run GUI script.
      iii. Select Configuration File or click cancel to generate a new one.
      iv. Click “Detect Port,” if necessary.
      v. Select Arduino Port.
      vi. Click “Connect.”
      vii. After connection is acknowledged click “Home.”

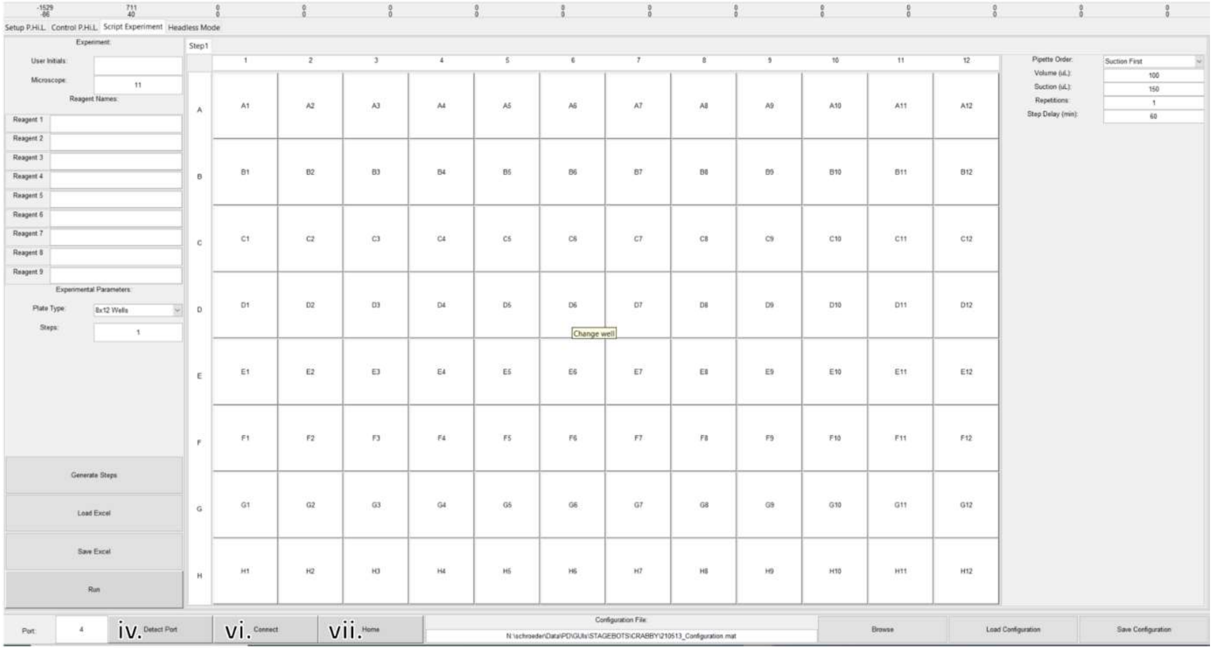

   b. **Calibrate Robot**
      i. Place a 96 well plate in the stage.
      ii. Move the robot to your desired wells.
      iii. For each well:
         1. When the pipet tips are above each well enter an estimated offset.
         2. Click “Go to Offset.”
         3. If you are satisfied with the adjustment click “Set offset”
         4. If you are not satisfied with the adjustment click “Go back” and enter new offset values.
      iv. Click any well.
      v. Click “Go to Well Bottom.”
      vi. Adjust the pipet height up or down.
      vii. When satisfied click “Set Well Bottom.”

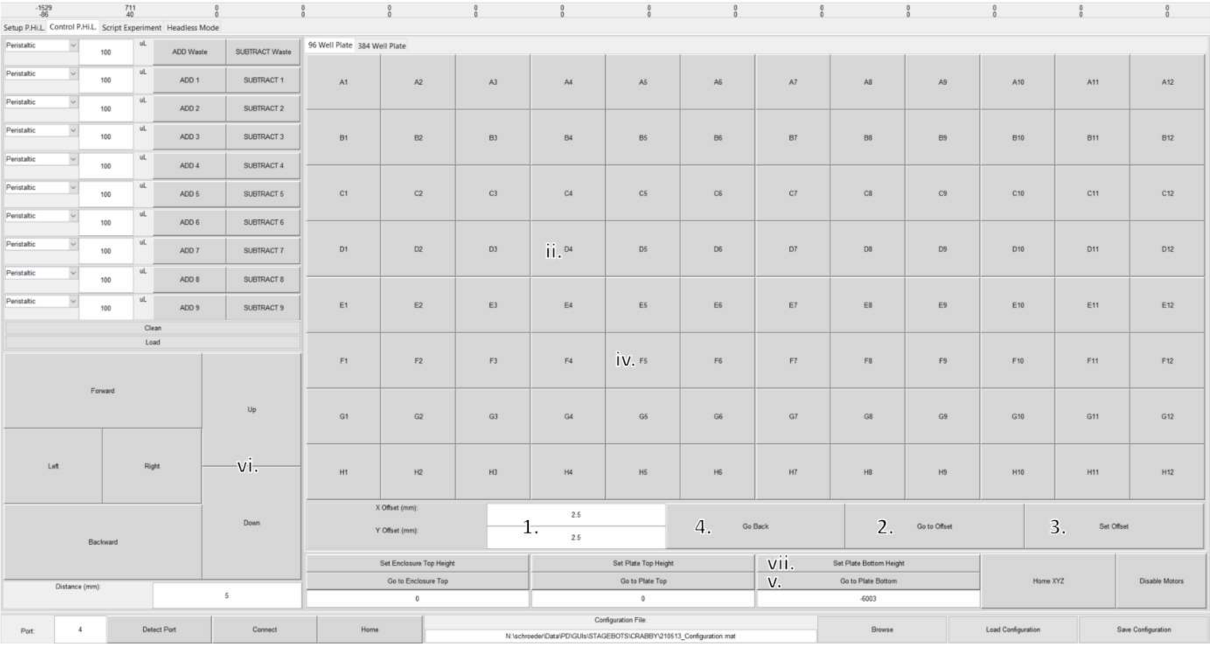

c. **Script experiment**

i. Enter your desired step count.
ii. Select your desired plate type.
iii. Click “Generate Steps.”
iv. Enter your fluid names in their assigned locations.
v. Enter your desired times between steps for each step.
vi. Enter your desired suction and addition volumes for each step.
vii. Click each well you wish the robot to modify and enter your desired ratios for each fluid for each step.
viii. Click “Save Excel.”
ix. Click “Run.”

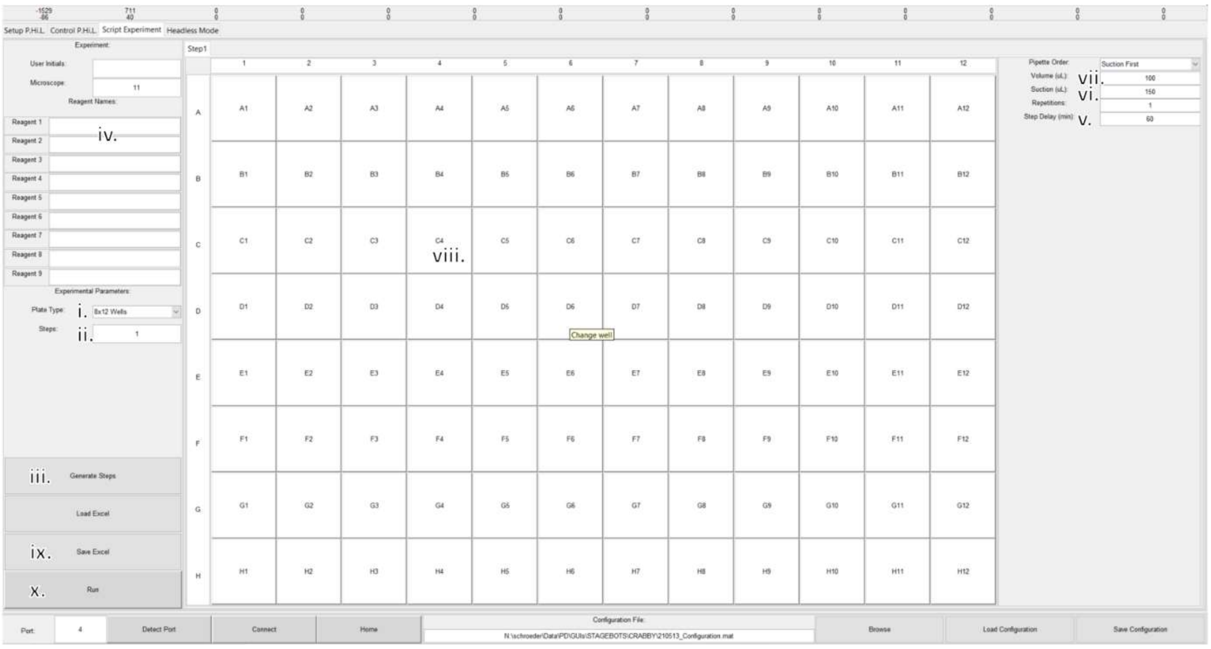

Supplement: Supplementary file 4 — Supplementary Software [file 41467_2022_30643_MOESM4_ESM.zip › PHIL-main/Operation_Instructions/PHIL_Operation_Instructions.pdf]
