# Supplementary material for: Open-source personal pipetting robots with live-cell incubation and microscopy compatibility
Source: Nat Commun. 2022 May 30;13:2999. doi: 10.1038/s41467-022-30643-7 (PMC9151679; doi:10.1038/s41467-022-30643-7)

Print all sets with:

- 3 walls
- .15 mm layer height

Print sets 1,2,4-10 with

- 20% infill

Print set 3 with:

- 100% infill

Set 1

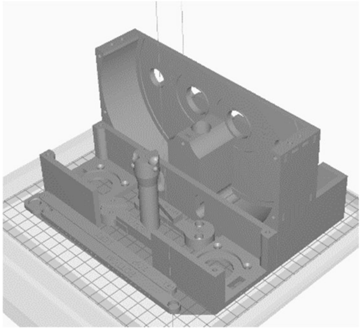

Set 2

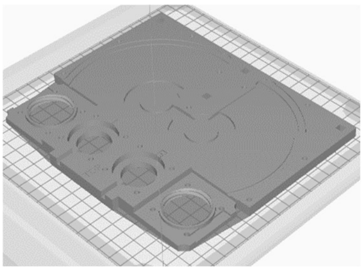

Set 3

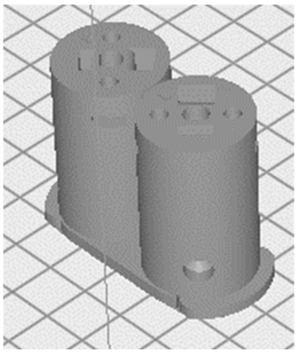

Set 4

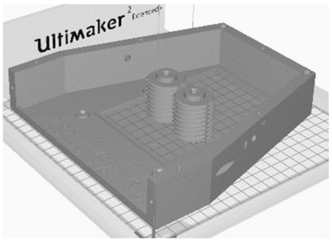

Set 5

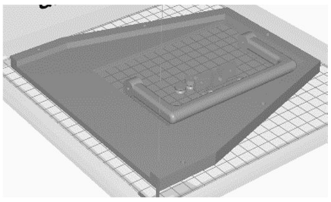

Set 6

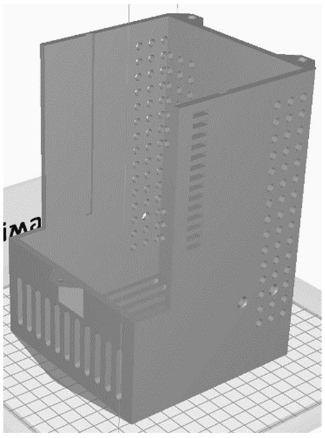

Set 7

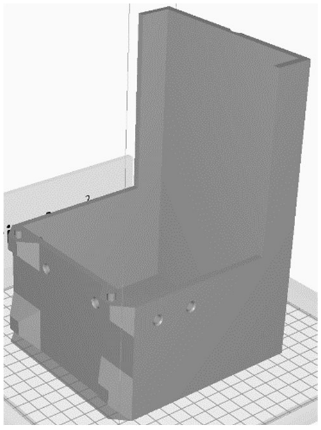

Set 8

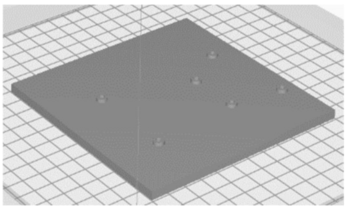

Set 9

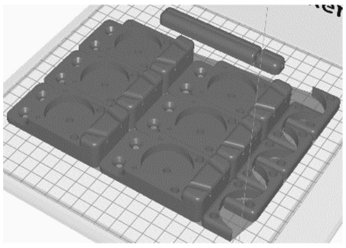

Set 10

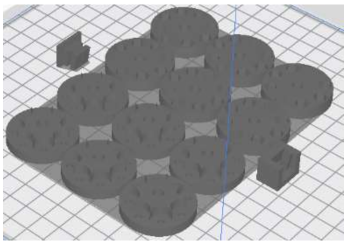

Supplement: Supplementary file 4 — Supplementary Software [file 41467_2022_30643_MOESM4_ESM.zip › PHIL-main/PHIL_Printable_Files/PHIL_Print_Instructions.pdf]
